# Gallocatechin-silver nanoparticles embedded in cotton gauze patches accelerated wound healing in diabetic rats by promoting proliferation and inhibiting apoptosis through the Wnt/β-catenin signaling pathway

**DOI:** 10.1371/journal.pone.0268505

**Published:** 2022-06-23

**Authors:** Vendidandala Nagarjuna Reddy, Shaik Nyamathulla, Khomaizon Abdul Kadir Pahirulzaman, Seri Intan Mokhtar, Nelli Giribabu, Visweswara Rao Pasupuleti

**Affiliations:** 1 Faculty of Agro Based Industry, University Malaysia Kelantan, Jeli Campus, Jeli, Kelantan, Malaysia; 2 Department of Pharmaceutical Technology, Faculty of Pharmacy, Universiti Malaya, Kuala Lumpur, Malaysia; 3 Department of Physiology, Faculty of Medicine, Universiti Malaya, Kuala Lumpur, Malaysia; 4 Department of Biomedical Sciences and Therapeutics, Faculty of Medicine and Health Sciences, Universiti Malaysia Sabah, Kota Kinabalu, Sabah, Malaysia; 5 Department of Biochemistry, Faculty of Medicine and Health Sciences, Abdurrab University, Pekanbaru, Riau, Indonesia; 6 Centre for International Relations and Research Collaborations, Reva University, Bangalore, Karnataka, India; Institute of Science, Banaras Hindu University, Varanasi, INDIA

## Abstract

**Background:** Diabetes mellitus is a chronic metabolic disorder characterized by elevated plasma glucose levels. It is often defined as a lifestyle disease having severe economic and physiological repercussions on the individual. One of the most prevalent clinical consequences of diabetes is the lagging pace of injury healing leading to chronic wounds, which still to date have limited treatment options. The objective of this research is to look into the wound healing capabilities of gallocatechin (GC) and silver nanoparticles (AgNPs) impregnated patches in diabetic rats. Experimental rats were dressed patches and the wound healing skin region was dissected at the end of the experiment for molecular analysis. The wound healing rate in diabetic rats dressed with CGP2 and CGP3 & silver sulfadiazine (AgS) patches were found to be high. While mRNA and immunofluorescence or immunohistochemistry assays reveal that Wnt3a and β-catenin levels were higher with Gsk-3β and c-fos levels were lower in diabetic rats dressed with in CGP2 and CGP3 as compared with diabetic rats dressed with DC+CGP1. Furthermore, apoptosis markers such as caspase-3, caspase-9, and Bax levels were reduced, whereas anti-apoptosis maker (Bcl-2) and proliferation marker (PCNA) levels were increased in diabetic rats dressed with CGP2 and CGP3 as compared with diabetic rats dressed with DC+CGP1. In conclusion, the results demonstrated that GC-AgNPs-CGP (CGP2 & CGP3) dressing on diabetes wound rats decreased changes in Wnt3a/β-catenin pathways, resulting in lower apoptosis and greater proliferation, so drastically improving diabetic wound healing.

## Introduction

Diabetes is a serious, chronic illness that occurs when the pancreas fails to produce enough insulin (a hormone that regulates blood sugar, or glucose) or when the body is unable to use the insulin that is produced efficiently. Diabetes is a major public health issue, and it is one of four priority noncommunicable diseases (NCDs) that world leaders have identified for action as it is associated with premature death and disability [1]. To reduce the occurrence of the manifestations, glucose-lowering strategies aimed at better control of DM metabolic disorders, as well as lifestyle change campaigns, are required. Long-term optimal glycemic control, however, is not always achieved in these patients. Diabetes has been steadily increasing in both the number of cases and its prevalence over the last few decades. Alarmingly, diabetes affected an estimated 422 million adults worldwide in 2014, up from 108 million in 1880 [2]. Since 1880, the global prevalence of diabetes (age-standardized) has nearly doubled, rising from 4.7 percent to 8.5 percent in the adult population. This is due to an increase in risk factors such as being overweight or obese. Diabetes prevalence has risen faster in low- and middle-income countries than in high-income countries over the last decade [3].

Chronic hyperglycemia caused by diabetes leads to several macrovascular (ischemic heart disease, stroke, and peripheral artery disease) and microvascular (neuropathy, nephropathy, and retinopathy) complications. In addition to various vascular implications, impaired wound healing is also a complication significantly affecting mobility, risk of bacterial infections, amputation and even death in some cases [4]. Diabetes causes impaired healing due to complex pathophysiology involving vascular, neuropathic, immune, and biochemical components. Hyperglycemia is associated with stiffer blood vessels, which causes slower circulation and microvascular dysfunction, resulting in decreased tissue oxygenation. Diabetes-related blood vessel changes account for decreased leukocyte migration into the wound, making it more susceptible to infection [5]. As a result, diabetic patients have a 15–25 percent lifetime risk of developing diabetic foot ulcers, with 40–80 percent becoming so infected that it involves the bone, resulting in osteomyelitis.

In general, wound healing is a very complex phenomenon that occurs when skin integrity is lost and consists of three phases: the first phase: inflammatory response (involving cytokines and growth factors); the second and third phases: Proliferation and Remodeling (leads to wound closure) are characterised by hypoxia and active proliferation of endothelial cells and subsequent mating [6]. Diabetes-related wound healing is characterised primarily by chronicization of inflammatory conditions, disruption of the angiogenic process, reduction of endothelial progenitor cells, and an imbalance in extracellular matrix regulation, as well as prompt incursion of neutrophils and macrophages driven by chemotactic chemokines that are especially elevated in diabetes. Infiltrating cells release inflammatory cytokines such as interleukin α (IL-α) and tumor necrosis factor α (TNF-α), which remain at high concentrations for longer periods of time, resulting in a prolonged inflammatory response [7]. Additionally, the Wnt3a/β-catenin regulated pathways are being explored to exploit its apoptosis proliferation potential already established functions like anti-cancer and fibrosis control. The Wnt signaling pathway regulates cell proliferation in the adult epidermis, which influences the rate and extent of skin wound healing. Using the Axin2-Cre lineage reporter rats, one study demonstrated that most of the basal epidermal layer requires Wnt/β-catenin signaling to proliferate, and that these same cells contribute significantly to wound healing without the need for a quiescent stem cell subpopulation.

Wnt3a/β-catenin signaling pathways in diabetes enhances the production of several growth factors involved in the initiation and maintenance of the healing process while reducing the inflammatory response [8]. Furthermore, current research in wound dressing indicates that dressings that provide a moist healing environment, such as calcium alginate, transparent films, or hydrocolloids, are associated with the quickest healing times. Furthermore, hydrocolloid and transparent film dressings were more likely than air exposure, xenografts, gauze, or calcium alginate dressings to result in a smooth stable epithelial surface [9, 10]. Much more recently, the combination of anti-oxidative compounds (catechins) and nanoparticles (silver and gold) is being pitched as a more effective approach for wound management in diabetics [11]. Gallocatechin have been established to improve the wound repair and regeneration and adding nanoparticles has improved its efficiency [12, 13]. This is due to the antimicrobial properties of the nanoparticles and the gallocatechin anti-oxidative traits which modulate the inflammatory response primarily [14, 15]. Thus, in this study, we assessed the efficacy and mechanism of gallocatechin-silver nanoparticle preparation impregnated cotton gauge patch as topical dressing on wound healing in Streptozotocin-induced diabetic mouse.

## Materials and methods

### Materials

Streptozotocin and Silver sulfadiazine (AgS) were procured from Sigma Company St. Louis, MO, USA respectively. Gallocatechin was procured from Chem Faces, Wuhan, China. Silver nanoparticles (AgNPs; <150 nm; Purity: 99%,) purchased from Chem Cruz, Heidelberg, Germany.

### Inducing diabetes in test animals

6 to 8 weeks old healthy Sprague Dawley (SD; 220 ± 30g) male rats were procured from University Malay animal house and maintained under controlled temperature (20 ± 4°C) and humidity (40 ± 10%) and maintained on standard diet. The study was approved by ethical committee, institutional animal ethics committee, Faculty of Medicine, Universiti Malaya, Kuala Lumpur, Malaysia (2019-210806/PHYSIO/R/GN). The induction of diabetics was described previously [16]. Briefly, overnight fasting rats were injected with nicotinamide (110 mg/kg bw; i.p) after 15 min, rats were injected with streptozotocin (STZ) at 55 mg/kg bw. After 3 days and the onset of diabetes was confirmed with fasting blood glucose levels [17]. The control group serves as non-diabetic animals for the study.

### Wound infliction and gallocatechin-silver nanoparticle (GC-AgNPs) patch application

As described in our previous study [18], after GC-AgNPs preparation (**S1 Fig**), impregnated in cotton gauze (**S2 Fig**) and evaluated (**S3 Fig**). Then 2cm wound was created on near back of neck region. After completely removing the blood, GC-AgNPs cotton gauge patchwith different treatments was applied (**S4 Fig**) to the rats to evaluate the wound healing efficiency and possible involved mechanism. Depending on the treatments the rats were further segregate into their respective groups as follows:

Group I: non-diabetic control (blank group) -treated with blank cotton gauze patch (NC-CGP1)
Group II: diabetic control—treated with blank cotton gauze patch (DC-CGP1)
Group III: diabetic rats—treated with 13.06μM GC-AgNPs cotton gauze patch (CGP2)
Group IV: diabetic rats—treated with 26.12 μM GC-AgNPs cotton gauze patch (CGP3)
Group V: diabetic rats (positive control)—treated with silver sulfadiazine (AgS) cotton gauze patch (CGP4)

After the dressing with CGP, all the individual rats were kept in separate holding cages. The wound healing attributes both superficial (continuously by visual observation) and histological were measured at the end of 15–18 days of the wound infliction and treated CGP application.

### Measurement of the wound area

The patches were removed and photographed at 3, 6, 9, 12, 15, and 18 days to determine the wound healing rate.

### Evaluation of wound healing and its mechanism

In order to elucidate the effect of GC-AgNPs-CGP on wound healing effectively, the wound healing traits at tissue and cellular level were measured using histophysiology and molecular studies involving staining techniques (Periodic acid-Schiff staining (PAS), immunofluorescence, immunohistochemistry) and antibody based fluorescent microscopic observations to detect induction of cellular signaling molecules involved [19].

### Histophysiology of the wound

After sacrifice the rats, the wound tissue was collected and kept in a 10% formalin and RNALater solution (Ambion, Austin, TX, USA) prior to histopathological studies and RNA extraction. To prepare tissue sections for microscopic observations, standard tissue preparation procedure was followed (formalin treatment to fix cellular and integrity, dehydration in increasing concentrations of ethanol, clearing with xylene, and embedding with paraffin wax and molded out). The molded-out tissue blocks were fed to a microtome to cut sections of 5μm thickness, which were then floated on to a clean glass slides using a warm water bath (30°C) and keep drying for one day.

### Periodic Acid Schiff (PAS) staining

The tissue sections (⁓ 5 μm thickness) were similarly de-paraffinized using several changes in xylene followed by hydration using a descending gradient of alcohol (100 to 70%) and finally with rinsing in milli Q water for 5 min. Subsequently the slides were flushed with periodic acid solution (for 5–10 min) then rinsed in tap water for few minutes and immersed in Schiff’s solution for 30 min and washed with hot water. Successively dipped in (modified Mayer’s) haematoxylin for 2 to 3 min and washed with distilled water and Blueing reagent was applied for 3 to 5 min. The slides were then dehydrated by rinsing in multiple changes of alcohol and mounted with a cover slip using Eco-Mount medium [20].

### Immunofluorescent and immunohistochemistry studies

To detect the presence and prevalence of different cellular proteins involved in cell proliferation and apoptosis during wound healing Immunofluorescent (Wnt3a/β-catenin, Bcl2, Caspase-3 and Bax) and Immunohistochemistry (Gsk-3β, C-FOS, Caspase-9 and PCNA) studies were carried out [21].

### Immunofluorescence staining (IF)

In this method the tissue sections were de-paraffined, hydrated and finally washed in milli Q water. And then, the sections were antigen retrieval was performed with citrate buffer (pH 6.0) by heated up to 95°C for 15 min and allowed to cool gradually (⁓ 20 min) followed by washing with milli Q water and incubated with 5 to 10% normal blocking serum in PBS for 1 h. This is followed by 1:500 primary antibodies treatment in serum of Blocking one solution (Nacalai Tesque, Kyoto, Japan) for overnight, after treatment the slides were washed with three changes of PBS (5 min each). Now fluorochrome conjugated secondary antibodies (1:1000; VectaFluor™ Duet Immunofluorescence Double Labeling Kit; DK-8828; Vector Laboratories, Burlingame, CA, USA) with 1.5 normal blocking serum was added and incubated 45 min in dark and washed thrice with PBS. Finally, mounted with 4′,6-diamidino-2-phenylindole (DAPI) mounting Media. A fluorescent microscope was used to examine slides and take photos at 400 x magnifications (Nikon Eclipse 80i microscope, SEO Enterprises Inc, Lakeland, FL, USA).

### Immunohistochemistry procedure (IHC)

After deparaffination, hydration and antigen retrieval tissue sections were incubated with 3% hydrogen peroxide (H_2_O_2_) for 30 min. (to neutralize endogenous peroxidases) and washed with three changes of PBS. The slides are then incubated in normal blocking serum for 1 hour then incubated with the target’s primary antibody (1:500 dilution) in normal blocking serum. After an overnight incubation, the slides were three times washed with PBS, then incubated with the secondary antibodies for 2 hours. Following washing the sections were incubated with diaminobenzene (DAB) (Nacalai Tesque, Japan) and stope the reaction with milli Q water. A counter stain, haematoxylin was applied for 30 to 40 seconds, then rinsed in water and dehydrated, cleared in xylene then mounted with a drop of mounting medium.

### Quantitative PCR or qPCR

The mRNA isolation and qPCR were carried out using the previously reported procedure [22]. To isolate total mRNA, wound tissues were kept in the Trizol reagent (Invitrogen, Carlsbad, CA). Then, using reverse transcription, 1 μg RNA was used to create cDNA (Thermo Scientific kit; Burlington, Canada). The cDNA samples were then used in an RT-PCR reaction (10ng of cDNA, 9 μl of qPCR Master Mix and 20 μl of respective primers) using SYBR® Premix Ex Taq™ (Tli RNaseH Plus) (Applied Biosystems). The relative gene expression was estimated using Ct values and 2^−ΔΔCT^ method. Table 1 lists the genes used in the investigation.

**Table 1:** List of primers used in this study.

Gene	Primer	Accession number
**Wnt3a**	F 5’-ACAGCAGCTTAATGACAGGGC-3	NM_001107005.2
	R 3’-CAGTGAGGAGTACTGGGGTCC-5’	
**Gsk-3β**	F 5’-ACTCTACCTGAACAGCCCCA-3’	NM_032080.1
	R 3’-AACGTGACCAGTGTTGCTGA-5’	
**β-catenin**	F 5’-GGCTAACATTCGCCAGTGGA -3	XM_008766691.2
	R 3’-TGCCACGTCAGCTGGTATAG-5’	
**c-fos**	F 5’-GCCTTCACCCTGCCTCTTC-3	XM_022197
	R 3’-GCTCCATGTTGCTAATGTTCTTGA-5’	
**Caspase-3**	F 5’-GAGCTTGGAACGCGAAGAAAA-3	NM_012922.2
	R 3’-TGCTTCCATGGATAGTCTTTGTTT-5’	
**Caspase-9**	F 5’-TCAGAACTGTCCCGTGAAGC-3	NM_031632.1
	R 3’-CTCCTCCAACCTGGAAGCTG-5’	
**Bax**	F 5’-GACACCTGAGCTGACCTTGG-3	NM_017059.2
	R 3’-AGTTCATCGCCAATTCGCCT-5’	
**Bcl-2**	F 5’- GACTGAGTACCTGAACCGGC-3	NM_016993.1
	R 3’- GCATGCTGGGGCCATATAGT-5’	
**β-actin**	F 5’-CTATGAGGGTTACGCGCTCC-3	EF156276.1
	R 3’-AGGTAGTCTGTCAGGTCCCG-5’	

### Statistical analysis

Data from the experiments were expressed as means ± SD. Data were analysed with one-way analysis of variance (ANOVA) by SPSS 18.0 (IBM, Armonk, NY, USA). Differences between groups of p≤0.05 were considered as a significant.

## Results

### Effect of GC-AgNPs CGP on wound closure rate

**Fig 1:**
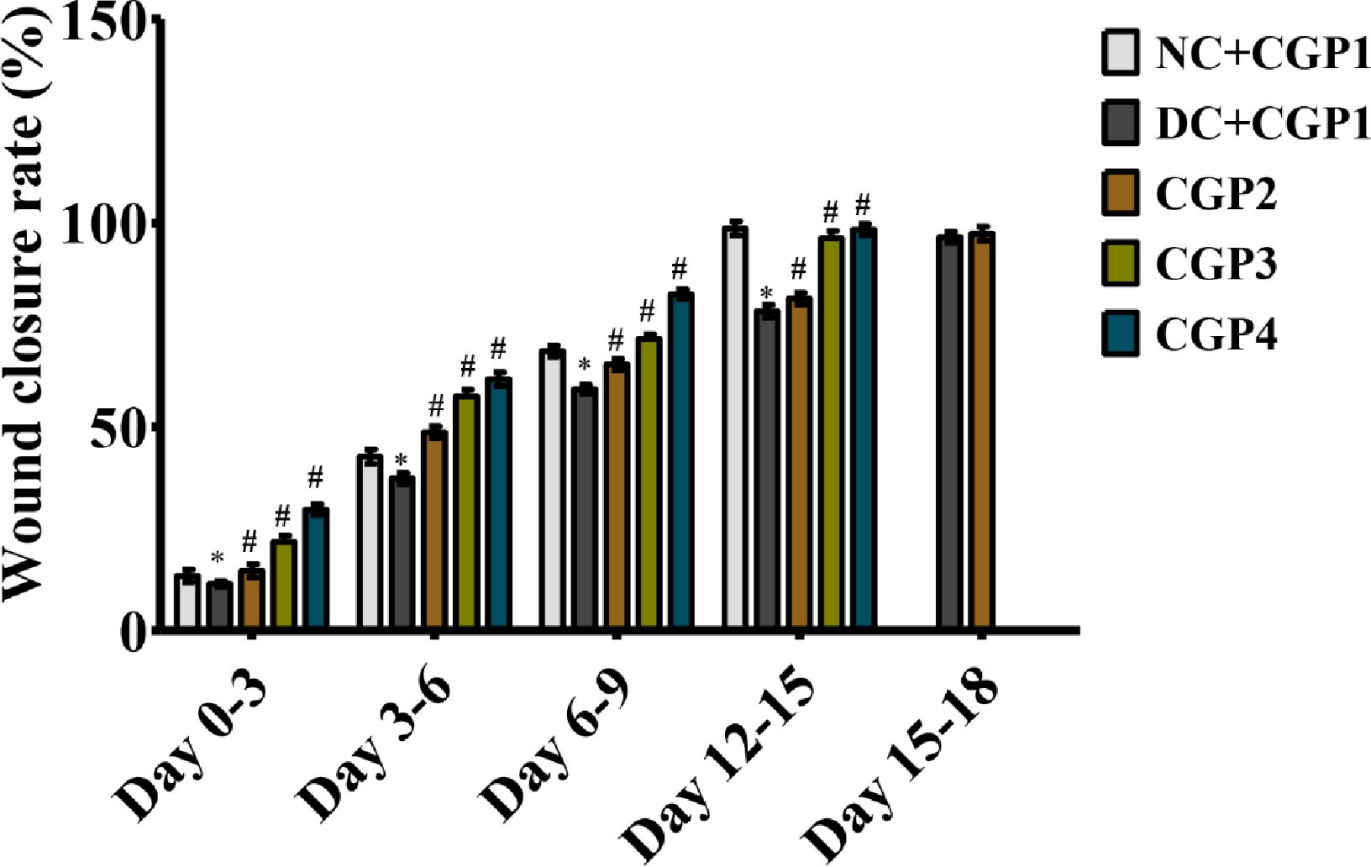
Effect of GC-AgNPs CGP dressing on wound healing contraction in diabetic rats. Data are expressed as mean ± S.D (n = 6). Bar graphs that do not share the same symbols are significantly different (p<0.05). NC+CGP1: Normal control dressed with blank cotton gauze; DC+CGP1: Diabetic control rats dressed with blank cotton gauze; CGP2: Diabetic rats dressed with 13.06 μM gallocatechin and silver nanoparticle (GC-AgNP) impregnated cotton gauze patches (DC+ 13.06 μM CGP2); CGP3: Diabetic rats dressed with 26.12 μM gallocatechin and silver nanoparticle (GC-AgNP) impregnated cotton gauze patches (DC+ 26.12 μM CGP3). CGP4: Diabetic rats dressed with silver sulfadiazine (DC+AgS) impregnated cotton gauze patches. Data were expressed as mean ± S.E.M (n = 6). *p < 0.05 compared to control, # p < 0.05 compared to DC.

Fig 1 depicts the rate of wound closure in experimental rats. The findings show that complete epithelization was accomplished in 18 days (CGP2-CGP4), while it required 18 days in diabetic rats dressed with blank cotton gauze patch (DC+CGP1). Furthermore, the percentage of wound contraction in control group on day 3, 6, 9 and 15 was 13.43%, 42.65%, 68.54% and 98.65% respectively. The percentage of wound contraction in DC+CGP1 was 11.43%, 37.35%, 59.16% and 78.32% respectively. The percentage of wound contraction in CGP2 was 14.65%, 48.65%, 65.27% and 81.43% respectively. Moreover, in CGP3 group the percentage of wound contraction on day 3, 6, 9 and 15 was 21.76%, 57.43%, 71.54% and 96.24% respectively. Also, in CGP4 group the percentage of wound contraction on day 3, 6, 9 and 15 was 29.65%, 61.67%, 82.50% and 98.35% respectively.

### Effect of GC-AgNPs CGP on histophysiology changes in skin wounds

**Fig 2:**
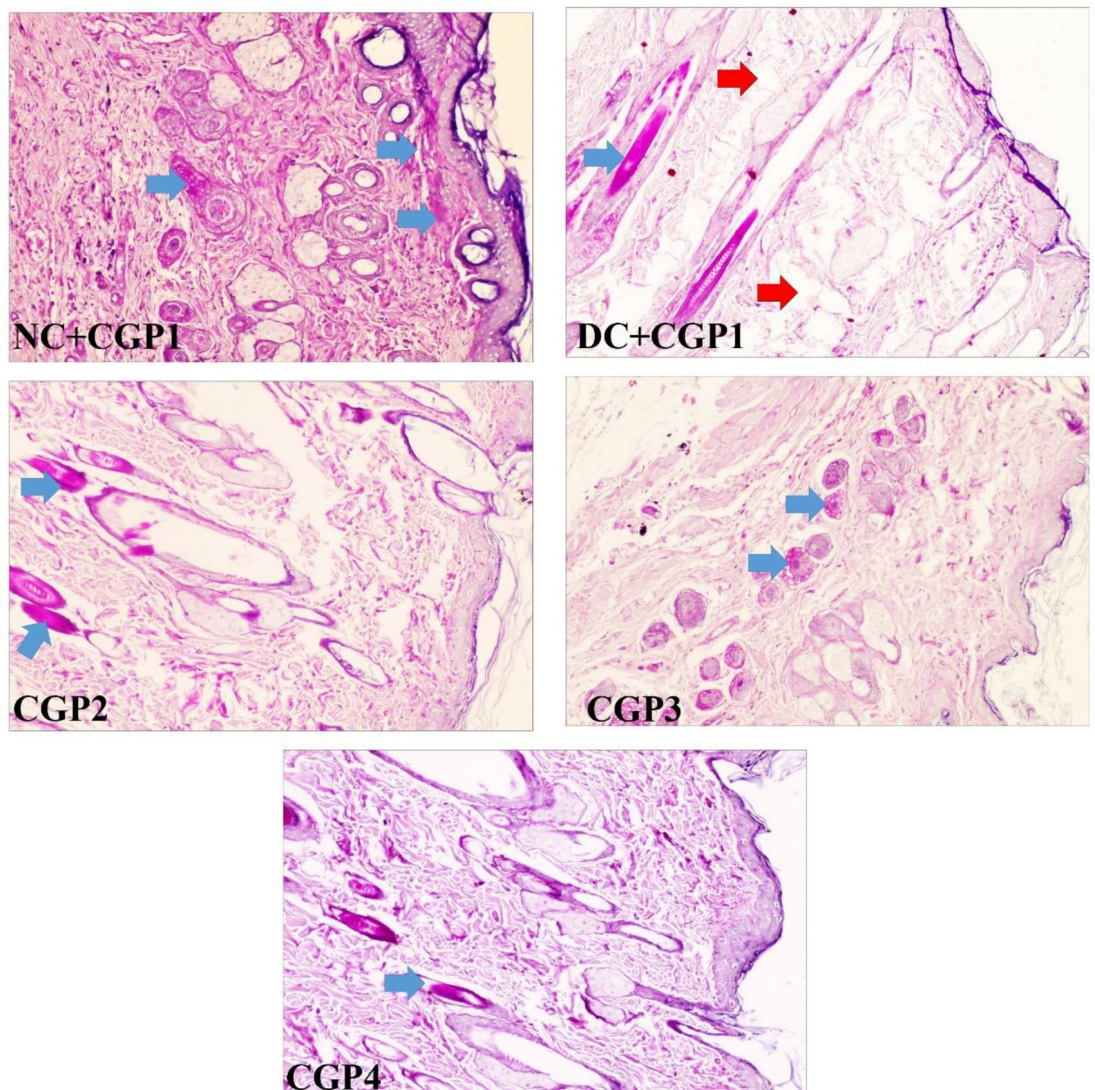
Effect of GC-AgNP CGPs dressing on histopathology in diabetic rats. Periodic Acid Schiff’s’ staining (PAS) rats wound tissue showing the cellular changes (glycogen & nuclear changes). Red arrows point towards the histopathological changes and blue arrow point towards glycogen distribution. NC+CGP1: Normal control dressed with blank cotton gauze; DC+CGP1: Diabetic control rats dressed with blank cotton gauze; CGP2: Diabetic rats dressed with 13.06 μM gallocatechin and silver nanoparticle (GC-AgNP) impregnated cotton gauze patches (DC+ 13.06 μM CGP2); CGP3: Diabetic rats dressed with 26.12 μM gallocatechin and silver nanoparticle (GC-AgNP) impregnated cotton gauze patches (DC+ 26.12 μM CGP3). CGP4: Diabetic rats dressed with silver sulfadiazine (DC+AgS) impregnated cotton gauze patches. Scale bar = 100 μm. Images are taken at 400x magnification.

Histochemical PAS staining showed that the normal control wounds (NC+CGP1) have highest glycogen content followed by SS treated (CGP4) diabetic wound rats. The GC-AgNPs (CGP2 & CGP3) also showed higher distribution of glycogen content in the excised/ wounded tissue, indicating it is potential to hasten the wound recovery process. In complete contrast to these observations, the diabetic control rat’s (DC+CGP1) wound tissue showed the lowest glycogen levels and possessed large unfilled voids. However, such voids are also present in SS and CGP2 treated rat’s wound tissue but other diabetic wound tissue were significantly low in CGP3 treated diabetic rats (Fig 2).

### Activation of Wnt/β-catenin signaling pathway promotes wound healing

**Fig 3:**
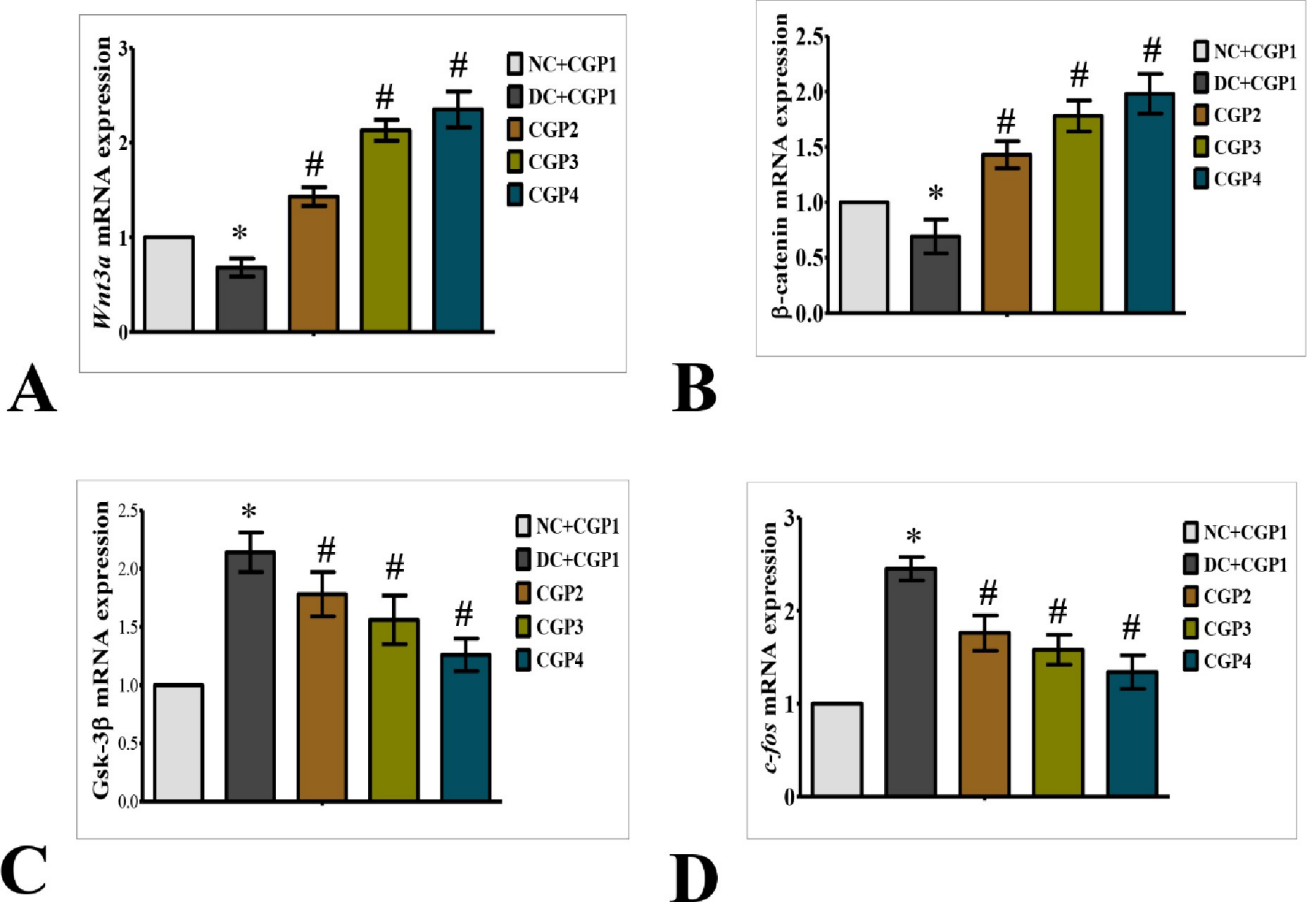
Effect of GC-AgNP CGPs dressing on Wnt signaling pathway in diabetic rats. Relative mRNA expression of (A) Wnt3a; (B) β-catenin; (C) Gsk-3β; (D) c-fos; NC+CGP1: Normal control dressed with blank cotton gauze; DC+CGP1: Diabetic control rats dressed with blank cotton gauze; CGP2: Diabetic rats dressed with 13.06 μM gallocatechin and silver nanoparticle (GC-AgNP) impregnated cotton gauze patches (DC+ 13.06 μM CGP2); CGP3: Diabetic rats dressed with 26.12 μM gallocatechin and silver nanoparticle (GC-AgNP) impregnated cotton gauze patches (DC+ 26.12 μM CGP3). CGP4: Diabetic rats dressed with silver sulfadiazine (DC+AgS) impregnated cotton gauze patches. Data were expressed as mean ± S.E.M (n = 6). *p < 0.05 compared to control, # p < 0.05 compared to DC.

**Fig 4:**
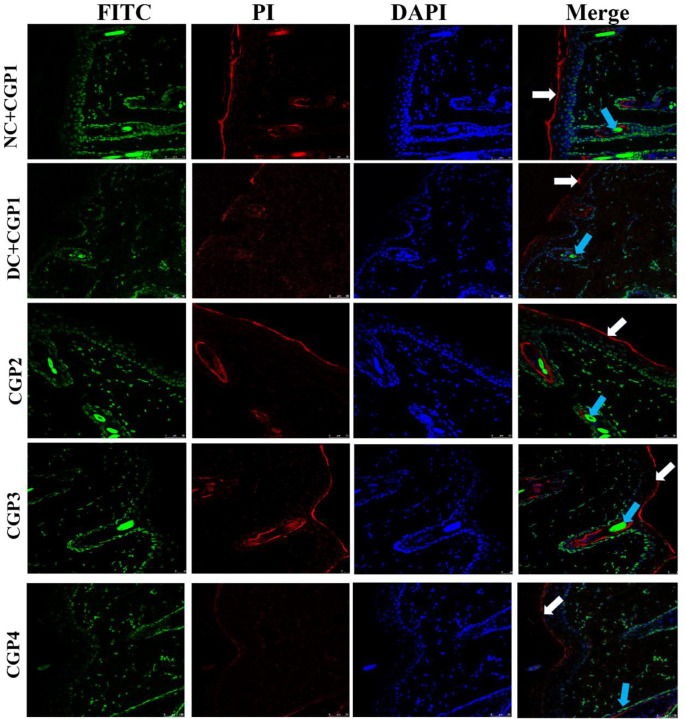
Effect of GC-AgNP CGPs dressing on Wnt3a and β-catenin in diabetic rats. Immunofluorescence double staining images showing co-localization of Wnt3a (red) and β-catenin (green). White arrows point towards the distribution of Wnt3a protein and blue arrow point towards the distribution of β-catenin. NC+CGP1: Normal control dressed with blank cotton gauze; DC+CGP1: Diabetic control rats dressed with blank cotton gauze; CGP2: Diabetic rats dressed with 13.06 μM gallocatechin and silver nanoparticle (GC-AgNP) impregnated cotton gauze patches (DC+ 13.06 μM CGP2); CGP3: Diabetic rats dressed with 26.12 μM gallocatechin and silver nanoparticle (GC-AgNP) impregnated cotton gauze patches (DC+ 26.12 μM CGP3). CGP4: Diabetic rats dressed with silver sulfadiazine (DC+AgS) impregnated cotton gauze patches. Data were expressed as mean ± S.E.M (n = 6). Scale bar = 100 μm.

**Fig 5:**
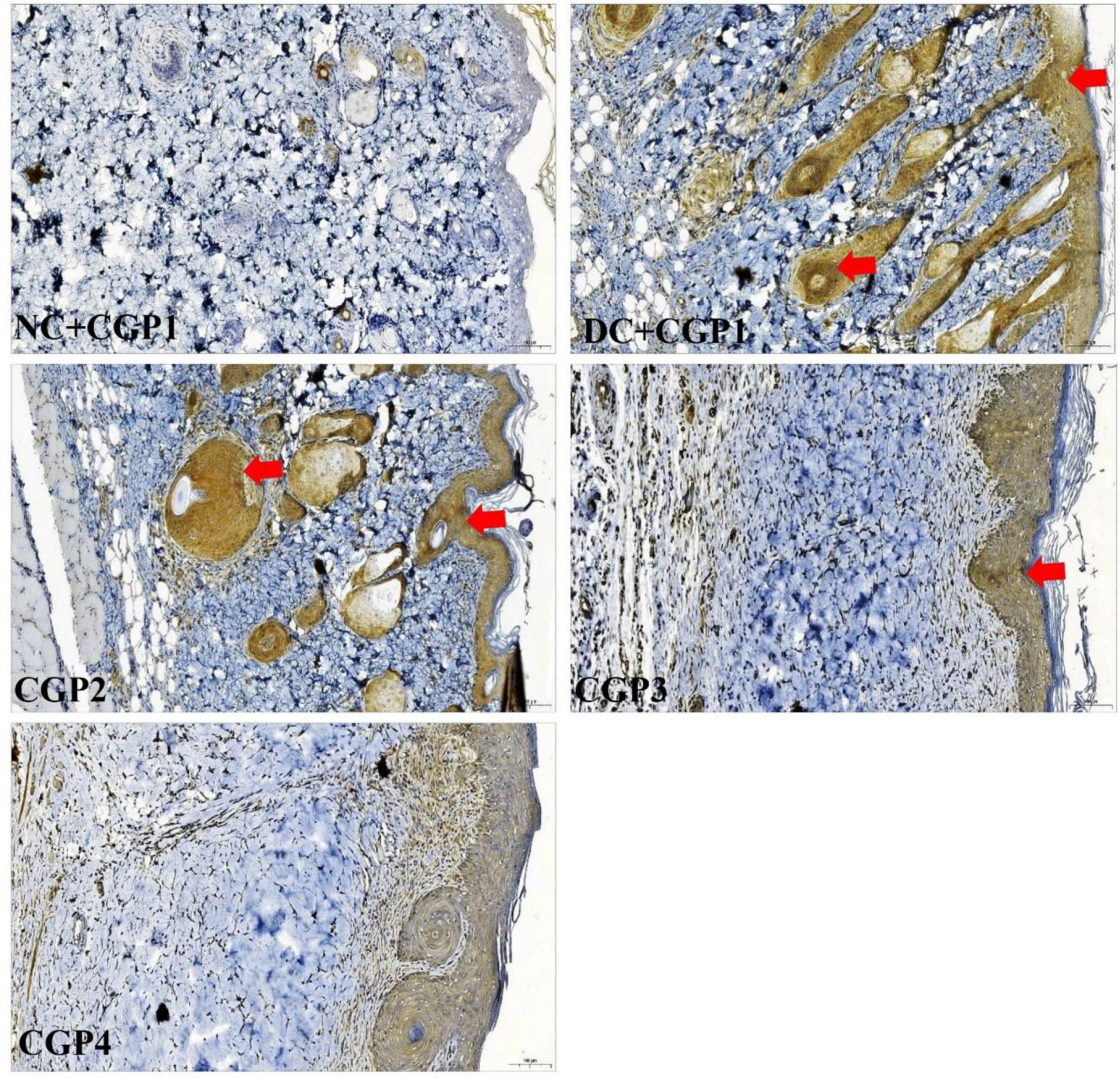
Effect of GC-AgNP CGPs dressing on Gsk-3β in diabetic rats. Immunohistochemistry staining of Gsk-3β (brown color). Red arrows point towards the distribution of Gsk-3β protein in brown color. NC+CGP1: Normal control dressed with blank cotton gauze; DC+CGP1: Diabetic control rats dressed with blank cotton gauze; CGP2: Diabetic rats dressed with 13.06 μM gallocatechin and silver nanoparticle (GC-AgNP) impregnated cotton gauze patches (DC+ 13.06 μM CGP2); CGP3: Diabetic rats dressed with 26.12 μM gallocatechin and silver nanoparticle (GC-AgNP) impregnated cotton gauze patches (DC+ 26.12 μM CGP3). CGP4: Diabetic rats dressed with silver sulfadiazine (DC+AgS) impregnated cotton gauze patches. Data were expressed as mean ± S.E.M (n = 6). *p < 0.05 compared to control, # *p* < 0.05 compared to DC. Scale bar = 100 μm.

Wnt3a/β-catenin, a mediator protein has opposing roles in keratinocytes and fibroblasts, inhibiting keratinocyte migration while activating fibroblast proliferation, implying that β-catenin could either inhibit or enhance the healing process. The Wnt/β-catenin signaling pathway in wound healing tissue is shown in Fig 3. The findings of q-PCR analyses revealed that diabetic rats dressed with CGP2 and CGP3 had increased mRNA levels of Wnt3a and β-catenin, but lower levels of Gsk-3β and c-fos. Immunofluorescence investigations further show that the distribution of Wnt3a and β-catenin proteins was lower in DC+CGP1 rats than in NC+CGP1. Wnt3a and β-catenin distribution was higher in diabetic animals clothed with CGP2 and CGP3 than in DC+CGP1 rats (Fig 4). Immunohistochemistry results also confirm that Gsk-3β (Fig 5) protein distribution was higher in DC+CGP1 dressed rats than NC+CGP1 rats. Furthermore, the distribution of Gsk-3β protein was reduced in CGP2 and CGP3-dressed rats compared to DC+CGP1-dressed rats.

**Fig 6:**
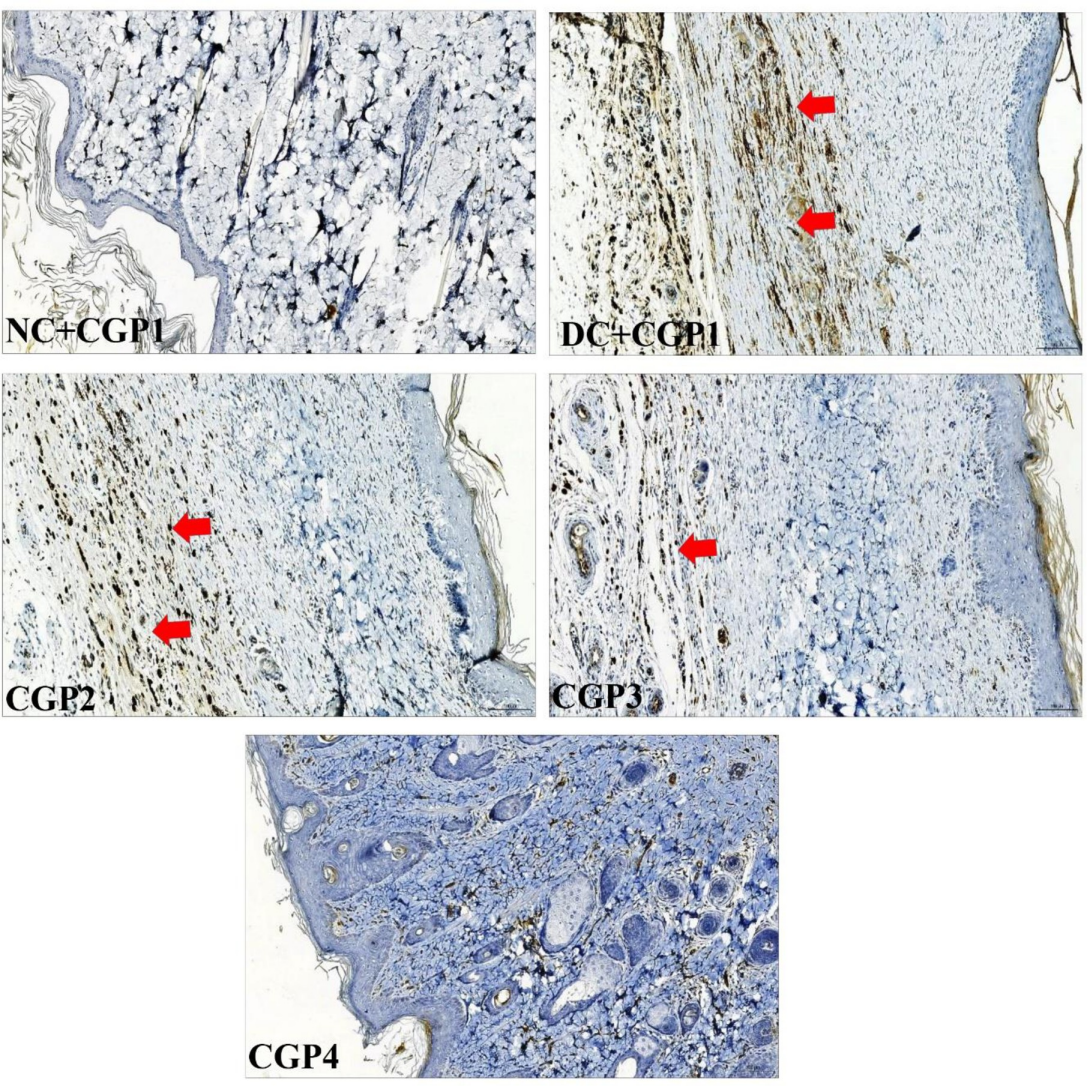
Effect of GC-AgNP CGPs dressing on c-fos in diabetic rats. **Immunohistochemistry staining of c-fos (brown color).** Red arrows point towards the distribution of c-fos protein in brown color. NC+CGP1: Normal control dressed with blank cotton gauze; DC+CGP1: Diabetic control rats dressed with blank cotton gauze; CGP2: Diabetic rats dressed with 13.06 μM gallocatechin and silver nanoparticle (GC-AgNP) impregnated cotton gauze patches (DC+ 13.06 μM CGP2); CGP3: Diabetic rats dressed with 26.12 μM gallocatechin and silver nanoparticle (GC-AgNP) impregnated cotton gauze patches (DC+ 26.12 μM CGP3). CGP4: Diabetic rats dressed with silver sulfadiazine (DC+AgS) impregnated cotton gauze patches. Data were expressed as mean ± S.E.M (n = 6). *p < 0.05 compared to control, # *p* < 0.05 compared to DC. Scale bar = 100 μm.

Immunohistochemistry results also confirm that c-fos (Fig 6) protein distribution was higher in DC+CGP1 dressed rats than NC+CGP1 rats. Furthermore, the distribution of c-fos proteins was reduced in CGP2 and CGP3-dressed rats compared to DC+CGP1-dressed rats. The distribution of c-fos protein was greater in DC+CGP1 dressed rats than in NC+CGP1 dressed rats (Fig 6). Furthermore, as compared to DC+CGP1-dressed rats, the distribution of c-fos proteins was reduced in CGP2 and CGP3-dressed rats.

### Effect of GC-AgNPs CGP on apoptosis signaling in skin wounds

**Fig 7:**
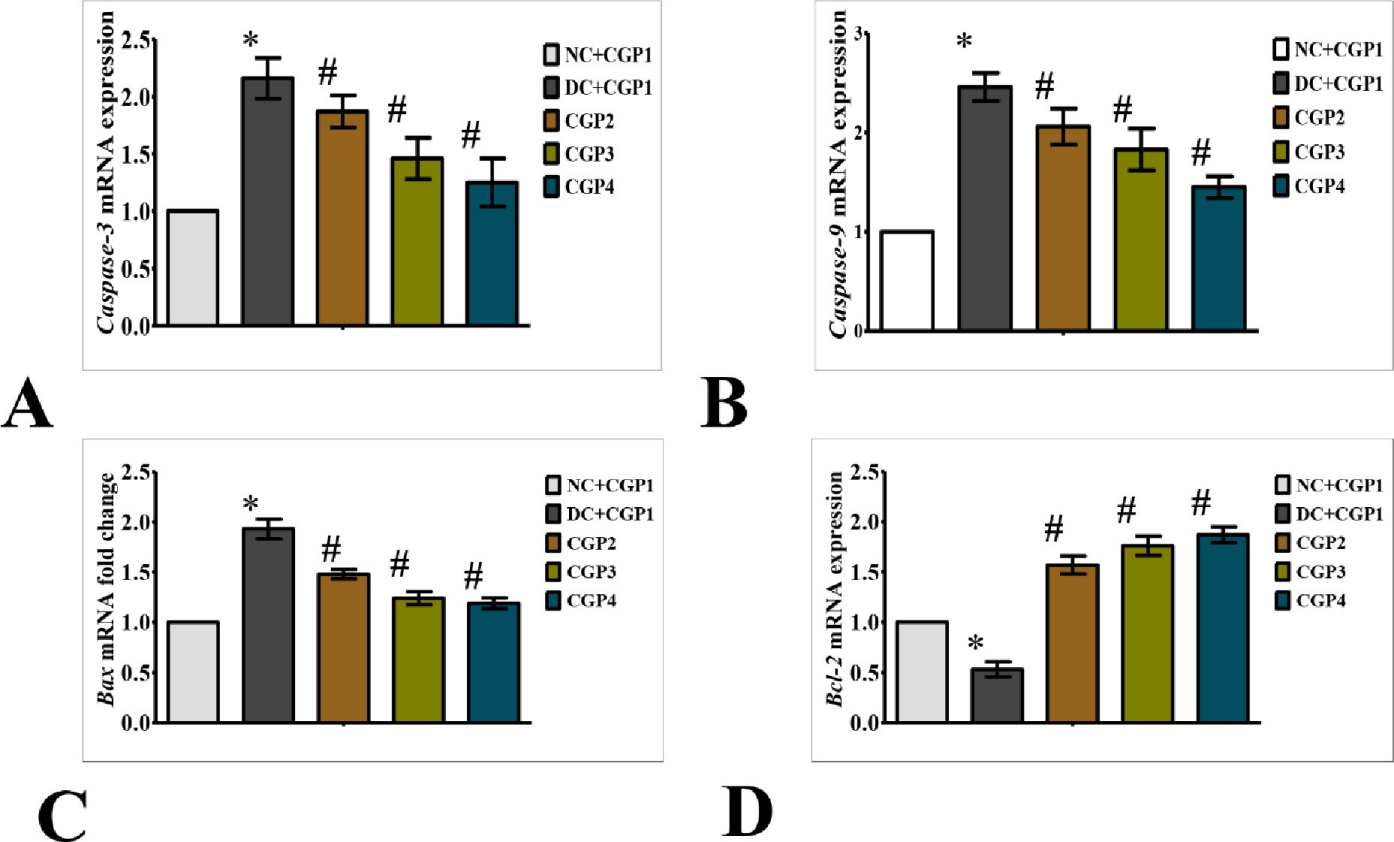
Effect of GC-AgNP CGPs dressing on apoptosis pathway in diabetic rats. Relative mRNA expression of (A) Caspase-3; (B) Caspase-9; (C) Bax; (D) Bcl-2; NC+CGP1: Normal control dressed with blank cotton gauze; DC+CGP1: Diabetic control rats dressed with blank cotton gauze; CGP2: Diabetic rats dressed with 13.06 μM gallocatechin and silver nanoparticle (GC-AgNP) impregnated cotton gauze patches (DC+ 13.06 μM CGP2); CGP3: Diabetic rats dressed with 26.12 μM gallocatechin and silver nanoparticle (GC-AgNP) impregnated cotton gauze patches (DC+ 26.12 μM CGP3). CGP4: Diabetic rats dressed with silver sulfadiazine (DC+AgS) impregnated cotton gauze patches. Data were expressed as mean ± S.E.M (n = 6). *p < 0.05 compared to control, # p < 0.05 compared to DC.

As shown in Fig 7, q-PCR was used to evaluate the mRNA of apoptotic markers in experimental wound healing rats. Apoptosis markers like caspase-3, caspase-9, and Bax mRNA levels were found to be greater in DC+CGP1 dressed rats than in NC+CGP1 dressed rats, whereas anti-apoptosis markers like Bcl-2 were found to be lower in DC+CGP1 dressed rats than NC+CGP1 rats.

**Fig 8:**
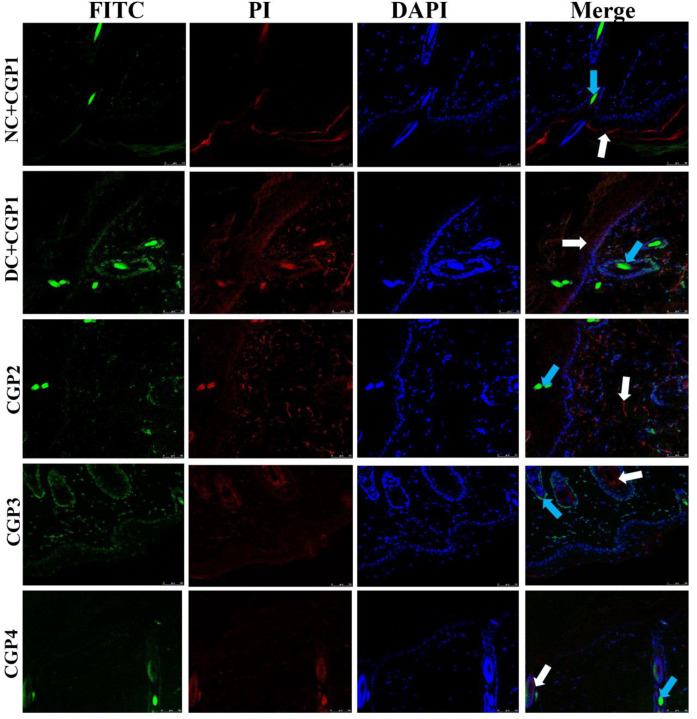
Effect of GC-AgNP CGPs dressing on caspase-3 and caspase-9 in diabetic rats. Immunofluorescence double staining images showing co-localization of caspase-3 (red) and caspase-9 (green). White arrows point towards the distribution of caspase-3 protein and blue arrow point towards the distribution of caspase-9. NC+CGP1: Normal control dressed with blank cotton gauze; DC+CGP1: Diabetic control rats dressed with blank cotton gauze; CGP2: Diabetic rats dressed with 13.06 μM gallocatechin and silver nanoparticle (GC-AgNP) impregnated cotton gauze patches (DC+ 13.06 μM CGP2); CGP3: Diabetic rats dressed with 26.12 μM gallocatechin and silver nanoparticle (GC-AgNP) impregnated cotton gauze patches (DC+ 26.12 μM CGP3). CGP4: Diabetic rats dressed with silver sulfadiazine (DC+AgS) impregnated cotton gauze patches. Data were expressed as mean ± S.E.M (n = 6). Scale bar = 100 μm.

The results of immunofluorescence double staining demonstrate that the distribution levels of caspase-3 and caspase-9 (Fig 8) were greater in DC+CGP1 dressed rats than in NC+CGP1 dressed rats. In contrast, diabetic rats dressed with CGP2 and CGP3 had a lower distribution of caspase-3 and caspase-9 when compared to diabetic rats dressed with DC+CGP1.

**Fig 9:**
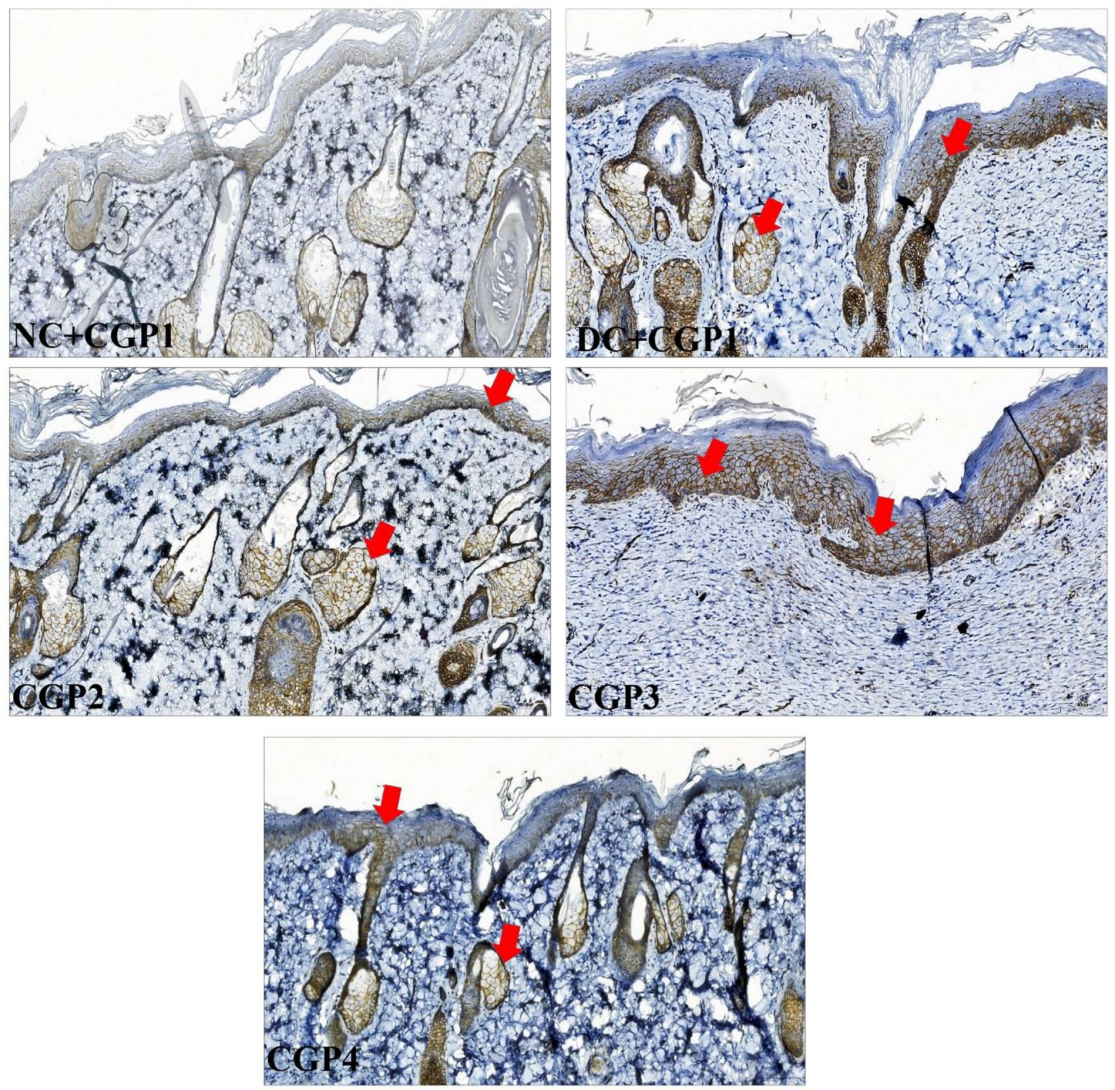
Effect of GC-AgNP CGPs dressing on BAX in diabetic rats. Immunohistochemistry staining of BAX (brown color). Red arrows point towards the distribution of BAX protein in brown color. NC+CGP1: Normal control dressed with blank cotton gauze; DC+CGP1: Diabetic control rats dressed with blank cotton gauze; CGP2: Diabetic rats dressed with 13.06 μM gallocatechin and silver nanoparticle (GC-AgNP) impregnated cotton gauze patches (DC+ 13.06 μM CGP2); CGP3: Diabetic rats dressed with 26.12 μM gallocatechin and silver nanoparticle (GC-AgNP) impregnated cotton gauze patches (DC+ 26.12 μM CGP3). CGP4: Diabetic rats dressed with silver sulfadiazine (DC+AgS) impregnated cotton gauze patches. Data were expressed as mean ± S.E.M (n = 6). *p < 0.05 compared to control, # *p* < 0.05 compared to DC. Scale bar = 100 μm.

**Fig 10:**
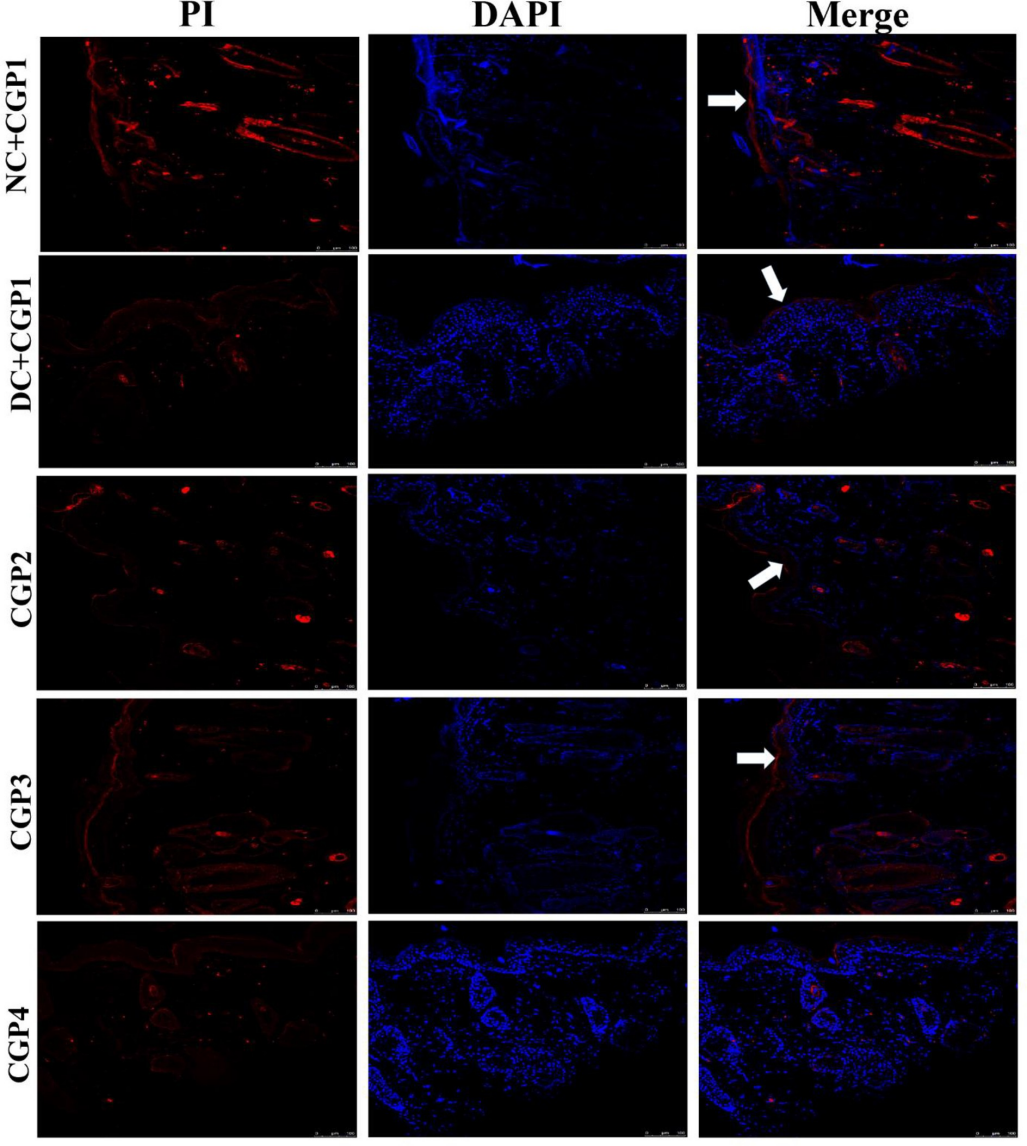
Effect of GC-AgNP CGPs dressing on BCL-2 in diabetic rats. Immunofluorescence images showing localization of BCL-2 (red). White arrows point towards the distribution of BCL-2 protein in red color. NC+CGP1: Normal control dressed with blank cotton gauze; DC+CGP1: Diabetic control rats dressed with blank cotton gauze; CGP2: Diabetic rats dressed with 13.06 μM gallocatechin and silver nanoparticle (GC-AgNP) impregnated cotton gauze patches (DC+ 13.06 μM CGP2); CGP3: Diabetic rats dressed with 26.12 μM gallocatechin and silver nanoparticle (GC-AgNP) impregnated cotton gauze patches (DC+ 26.12 μM CGP3). CGP4: Diabetic rats dressed with silver sulfadiazine (DC+AgS) impregnated cotton gauze patches. Data were expressed as mean ± S.E.M (n = 6). Scale bar = 100 μm.

According to immunohistochemistry, the BAX protein was highly distributed in DC+CGP1 dressed rats than in NC+CGP1 dressed rats (Fig 9). Diabetic rats dressed in CGP2 and CGP3 showed lower BAX protein distribution than diabetic rats dressed with DC+CGP1. Besides, immunofluorescence results also confirm that BCL-2 protein was lower distributed in DC+CGP1 dressed rats than in NC+CGP1 dressed rats (Fig 10). Diabetic rats dressed in CGP2 and CGP3 showed higher BCL-2 protein distribution than diabetic rats dressed with DC+CGP1.

### Effect of GC-AgNPs CGP on proliferation marker in skin wounds

**Fig 11:**
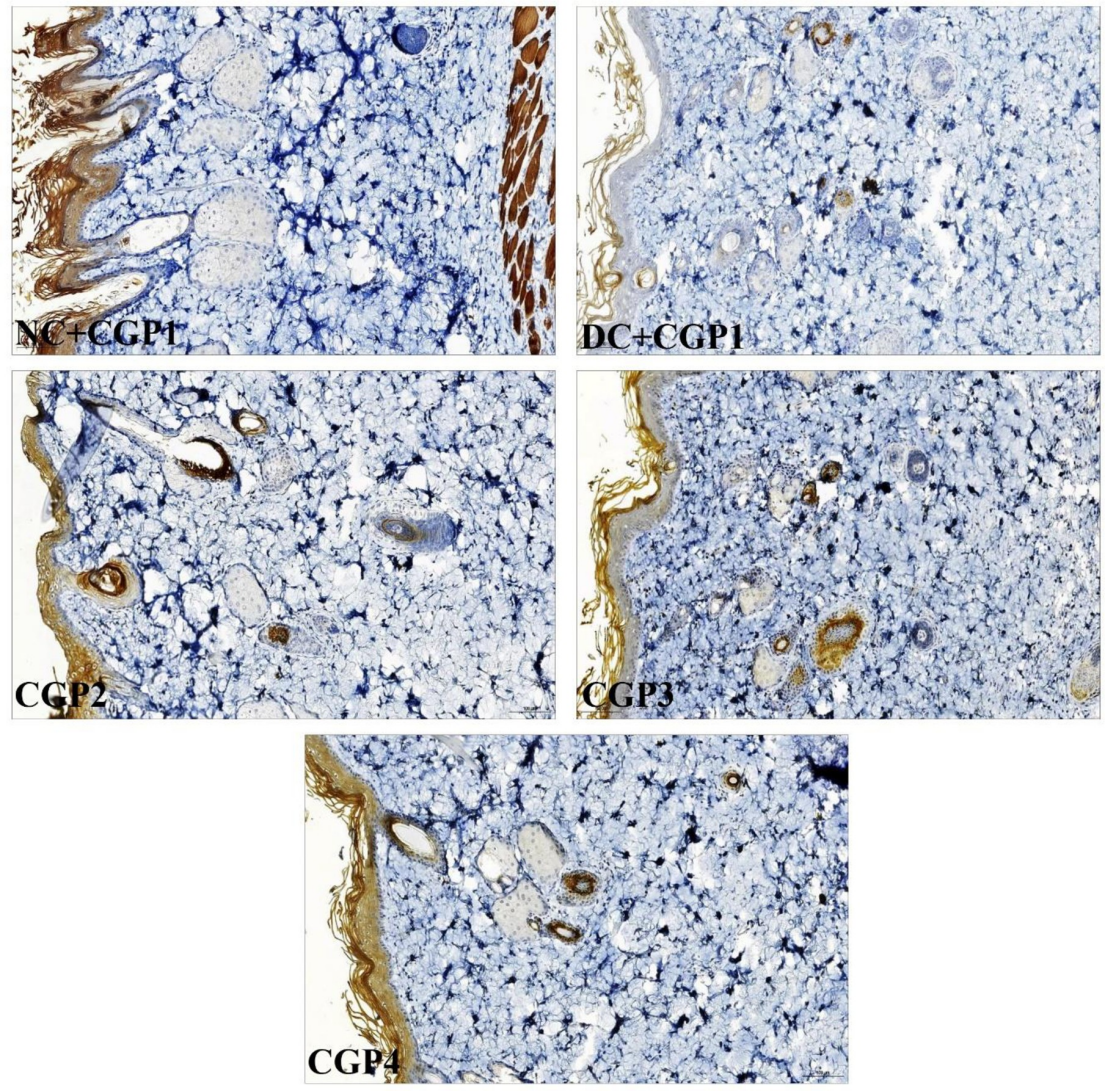
Effect of GC-AgNP CGPs dressing on PCNA in diabetic rats. Immunohistochemistry staining of PCNA (brown color). Red arrows point towards the distribution of PCNA protein in brown color. NC+CGP1: Normal control dressed with blank cotton gauze; DC+CGP1: Diabetic control rats dressed with blank cotton gauze; CGP2: Diabetic rats dressed with 13.06 μM gallocatechin and silver nanoparticle (GC-AgNP) impregnated cotton gauze patches (DC+ 13.06 μM CGP2); CGP3: Diabetic rats dressed with 26.12 μM gallocatechin and silver nanoparticle (GC-AgNP) impregnated cotton gauze patches (DC+ 26.12 μM CGP3). CGP4: Diabetic rats dressed with silver sulfadiazine (DC+AgS) impregnated cotton gauze patches. Data were expressed as mean ± S.E.M (n = 6). *p < 0.05 compared to control, # *p* < 0.05 compared to DC. Scale bar = 100 μm.

When diabetic rats were dressed with DC+CGP1, the distribution of proliferative markers such as PCNA was reduced compared to normal rats treated with NC+CGP1 (Fig 11). Furthermore, when diabetic rats were dressed with CGP2 and CGP3, the distribution of PCNA was observed to be greater than in diabetic rats treated with DC+CGP1, indicating that these treatments were more effective.

## Discussion

Wound healing is a complex cellular phenomenon that involves numerous factors and pathways, as well as a dynamic process of replacing devitalized and missing cellular structures and tissue layers. Wound healing can be divided into three or four distinct stages: inflammatory, proliferation, and remodelling [23]. Some researchers prefer hemostasis as the first phase, making the wound healing process a four-phase concept with hemostasis, inflammation, proliferation, and remodelling [23]. Any change or disruption in the normal functioning of signalling molecules regulating these wound healing phases will result in slower wound closure or the development of chronic wounds, particularly in conditions such as diabetes, where hyperglycemia is a challenge for normal physiological functions of the body [24]. In recent years, researchers have concentrated their efforts on developing natural active compounds that may heal skin wounds [25, 26]. In this study, we developed an effective diabetic rat wound model, allowing them to investigate the likely mechanism by which gallocatechin-silver nanoparticles accelerate wound healing. Gallocatechin-silver nanoparticles were proven to considerably help in wound healing in a series of experiments [18, 27].

Additionally, several experimental investigations have shown that the Wnt family is involved in a variety of biological processes, including cell proliferation, apoptosis and differentiation [28]. Additionally, it promotes angiogenesis and epithelial remodelling in wounds, as well as affects wound healing. As a result, we explored the role of the Wnt/β-catenin pathways in wound healing. In diabetic rats, Wnt signaling is also critical in wound healing. We examined Wnt signalling genes such as Wnt3a, β-catenin, GSK3β, and c-fos at both the mRNA and protein levels in this work. When β-catenin accumulates, the Wnt signalling pathway is stimulated [29]. Furthermore, Wnt3a and β-catenin expression levels are much lower in diabetic rats wound healing regions. Lower levels of Wnt3a and β-catenin expression may have impaired the diabetic rat ability to proliferate. Generally, GSK3β is considered to be a Wnt signalling inhibitor [30]. GSK3β activation results in β-catenin degradation, which decreases β-catenin expression. c-fos is a gene that promotes cell proliferation and differentiation in response to external stimuli such as wound healing [31]. According to our q-PCR data and immuno data, gallocatechin-silver nanoparticles increased the expression of Wnt3a, as well as β-catenin and decrease the GSK3β in wound area of diabetic rats applied with patches. These data suggest that CGP2 and CGP3 may increase Wnt3a signaling during wound healing. Similarly, other catechins, such as (-) epigallocatechin, activate the WNT/β-catenin pathway, causing β-catenin to be up-regulated, which subsequently causes essential to down-regulate the GSK3β and c-Fos [32].

In addition to the above mechanism, gallocatechin-silver nanoparticle patches (CGP2 & CGP3) were shown to improve wound healing by reducing skin cell death through apoptosis inhibition in diabetes rats. When diabetic rats were dressed with CGP2 and CGP3 patches, reduction in the expression and distribution of pro-apoptosis proteins like caspase-3, caspase-9, and Bax, as well as an increase in the expression and distribution of anti-apoptosis proteins like Bcl-2. According to these results, CGP3 dressed with diabetic rats decreases pro-apoptosis protein production while increasing anti-apoptosis protein expression. Finally, our research showed a significant decrease in proliferative activity in diabetic wounds, as shown by lower levels of PCNA proteins. CGP2 and CGP3-applied diabetic rats had increased PCNA levels, which improves proliferation. This implies that the patch action of gallocatechin-silver nanoparticles includes not only the genomic pathway, but also the non-genomic pathway. Similar to our study, (+)- catechin protects against oxidative stress-induced cell death in fibroblast, it has potential as a therapeutic agent for the prevention of skin aging [33]. All these regularities suggest the gallocatechin is efficient in normalising the normal and diabetic wound healing mechanism which is severely disrupted in diabetes and the gallocatechin silver nanoparticle preparation suggestiveness of its efficacy[34, 35].

## Conclusion

Our results demonstrate that the molecular mechanisms behind gallocatechin-silver nanoparticle patches on wound healing enhancement are mediated by GSK3β-mediated Wnt/ β-catenin signalling. As a result of the enhanced β-catenin signalling, it leads to decreased apoptosis and increased cellular proliferation during the wound healing process.

## Supporting information

S1 Fig Preparation of GC-AgNPs and AgS in chitosan solution. (TIF)

S2 Fig Preparation of GC-AgNPs and AgS impregnated cotton gauze. (TIF)

S3 Fig Evaluation of prepared GC-AgNPs and AgS impregnated cotton gauze. (TIF)

S4 Fig Apply of GC-AgNPs and AgS impregnated cotton gauzes on experimental rats. (TIF)
