# Supplementary figures and images for: Gallocatechin-silver nanoparticles embedded in cotton gauze patches accelerated wound healing in diabetic rats by promoting proliferation and inhibiting apoptosis through the Wnt/β-catenin signaling pathway
Source: PLoS One. 2022 Jun 23;17(6):e0268505. doi: 10.1371/journal.pone.0268505 (PMC9223326; doi:10.1371/journal.pone.0268505)

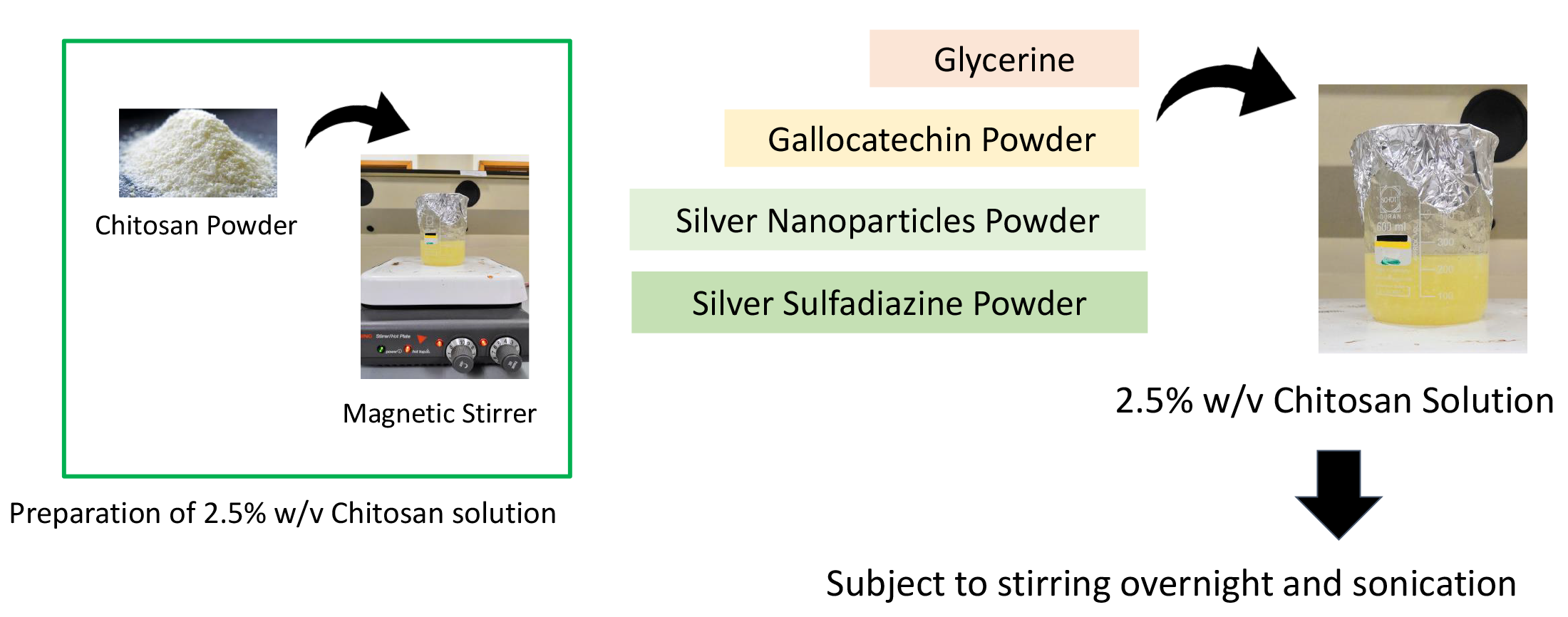

Supplement: S1 Fig — (TIF) [file pone.0268505.s001.tif]

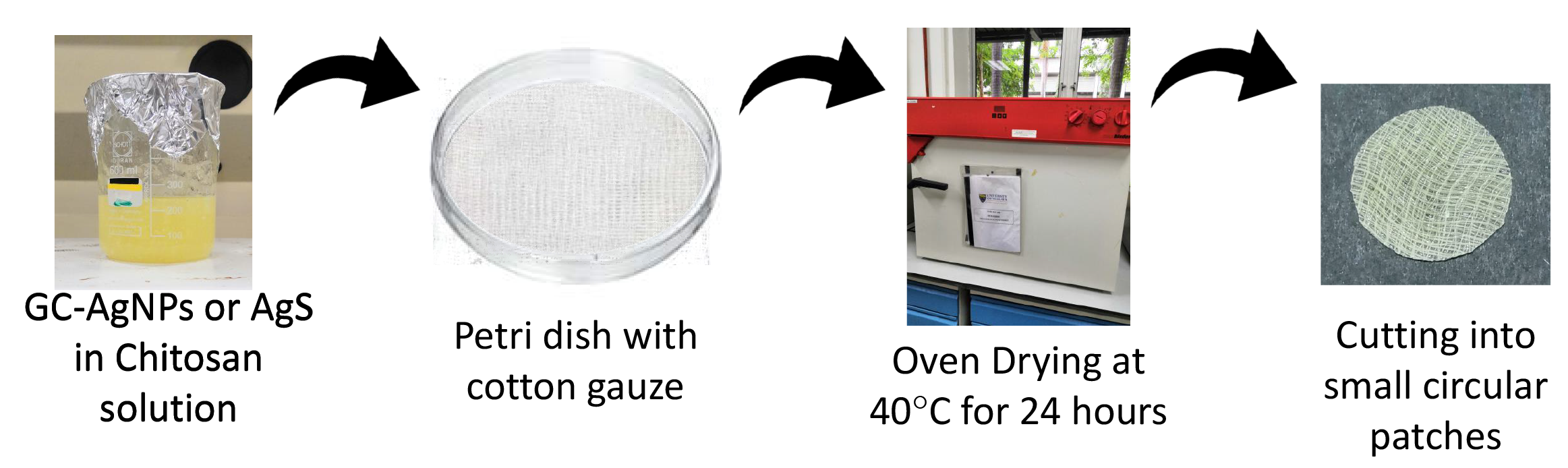

Supplement: S2 Fig — (TIF) [file pone.0268505.s002.tif]

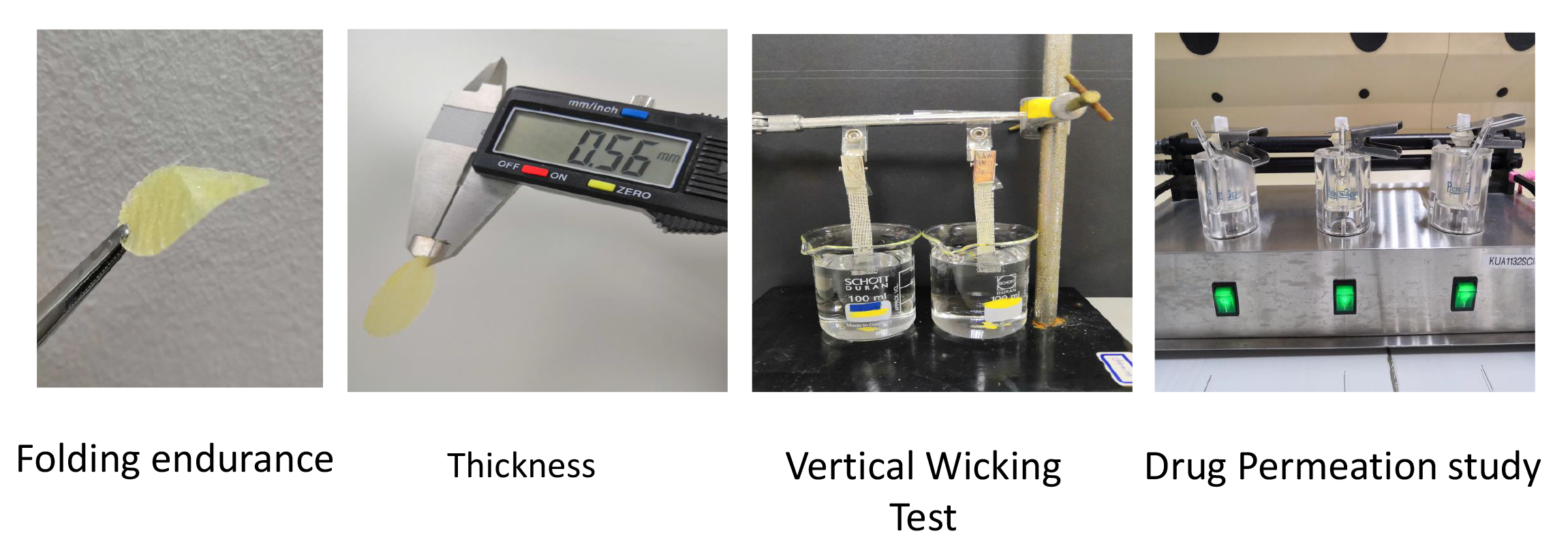

Supplement: S3 Fig — (TIF) [file pone.0268505.s003.tif]

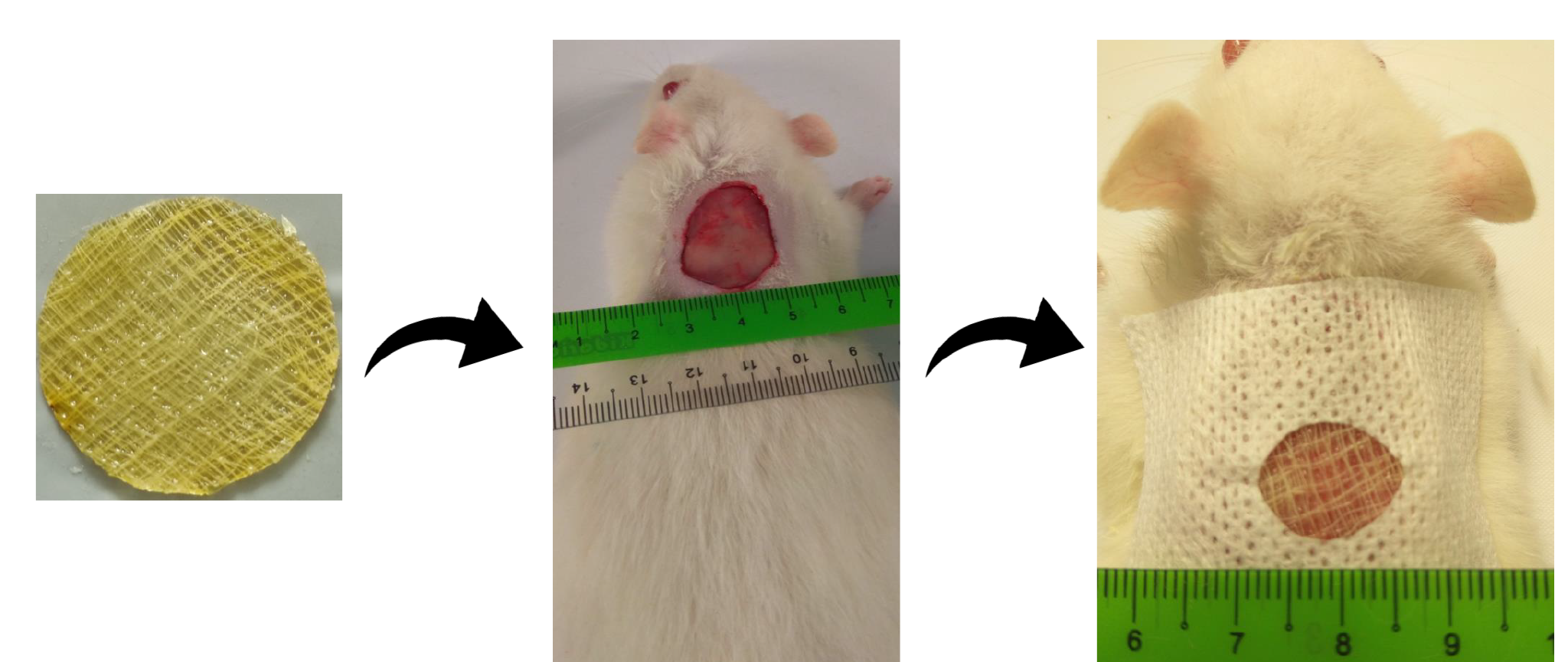

Supplement: S4 Fig — (TIF) [file pone.0268505.s004.tif]
